# Pricing strategies of the tobacco companies in response to cigarette excise tax increases in Montenegro

**DOI:** 10.1371/journal.pone.0335670

**Published:** 2026-06-02

**Authors:** Mirjana Čizmović, Ana Mugoša, Milica Vukčević, Violeta Vulović

**Affiliations:** 1 Department of Financial Management, Faculty of Economics and Business, Mediterranean University, Podgorica, Montenegro; 2 Institute for Socio-economic Analysis, Podgorica, Montenegro; 3 Department of Finance, Faculty of Economics, University of Montenegro, Podgorica, Montenegro; 4 Department of Finance and Accounting, Faculty of Economics, University of Montenegro, Podgorica, Montenegro; 5 Unit of Fiscal Policy and Sustainable Growth, World Bank, Washington DC, United States of America; Southern Illinois University Carbondale, UNITED STATES OF AMERICA

## Abstract

This study examines the impact of excise tax increases on cigarette prices in Montenegro, offering insights into the tobacco industry’s pricing strategies. Using both panel quantile regression and fixed-effects models, the research estimates excise tax pass-through to cigarette prices across different price ranges and market segments. The analysis is based on monthly price data from 2010 to 2022 for 269 cigarette brands. The findings reveal partial tax pass-through for lower-priced brands, while premium brands experience over-shifting, meaning tax increases are more than fully passed on to consumers. Slim cigarettes remain relatively affordable, as their prices never fully reflect tax increases. This pricing strategy allows the industry to maintain a substantial price gap between premium and low-cost cigarettes while sustaining profitability. Industry-driven cross-price subsidies for low-cost cigarettes undermine the intended impact of excise tax increases by maintaining their affordability and consumption. This highlights the need for comprehensive reforms in Montenegro’s tobacco tax policy to ensure effective tobacco control.

## Introduction

Tobacco use remains one of the leading causes of preventable diseases and mortality worldwide. By 2030, it is projected to account for an estimated 10 million annual deaths, with over two-thirds occurring in low-and middle-income countries [1]. Montenegro is no exception to this global trend, with an alarmingly high smoking prevalence. According to the World Health Organization (WHO) database, the global prevalence of tobacco use among adults (aged 15 years and older) was 21.1% in 2020 [2], while the estimated prevalence for Montenegro in the same year was 36.9% [3]. According to a Survey on tobacco consumption in Southeastern European countries [4], prevalence is even higher, where 40.1% of adults were smokers in 2020, and what is even more concerning, 20% had started smoking before the age of 15, which reflects a severe early-age initiation issue. With an average daily consumption of 19.7 cigarettes and a lack of effective cessation programs, most smokers in Montenegro find it challenging to quit [4].

Apart from the health consequences, smoking also imposes a significant economic burden. In Montenegro, low-income families allocate approximately 5.6% of their budget to tobacco, typically at the expense of basic necessities [5]. At the national level, the total economic cost of tobacco use was estimated at €307 million in 2020 – comprising direct medical expenses, lost productivity, and the economic cost of tobacco-attributable death – equivalent to a substantial 7.3% of the country’s Gross Domestic Product (GDP) [6]. Addressing this issue demands strong tobacco control to decrease health and economic expenses.

Effective implementation of key WHO Framework Convention on Tobacco Control measures-such as increased tobacco taxation, bans on smoking in public areas, and public awareness campaigns-has the potential to save over 6,700 lives and prevent €673 million in economic costs of tobacco use by 2037 [6]. Tobacco control would also enable Montenegro to move towards Sustainable Development Goal (SDG) target 3.4 of a one-third reduction in premature non-communicable disease deaths by 2030.

Among all tobacco control strategies, increasing cigarette prices through enhanced taxes is widely recognized as the most powerful approach for lowering prevalence and consumption. Extensive economic studies confirm that raising cigarette taxes and prices results in reductions in both adult and youth smoking levels [7–8]. Evidence from Montenegro supports this, with an estimated own-price elasticity of cigarette demand of −0.88 [9]. Montenegro employs a mixed excise tax framework for cigarettes, incorporating an ad valorem tax (assessed as a percentage of retail prices) along with a specific excise tax (a set amount per unit). Over the past two decades, excise taxes have been gradually increased through annual or semi-annual adjustments alongside other tobacco control measures. With the specific excise of €53.5 per 1,000 cigarettes and an ad valorem tax of 24.5 percent of the retail price starting in January 2025, Montenegro complies with the European Union Tobacco Products Directive’s minimum requirement of €90 per 1,000 cigarettes. However, despite these tax increases and other tobacco control efforts, cigarette prices remain low compared to the average prices in the European Union (EU) [10]. Coupled with recent fiscal expansion policies, this has led to increased cigarette affordability, undermining the intended impact of taxation on consumption reduction.

The effect of tobacco taxation relies on how much of the tax increase is passed on to consumer prices [11–12]. However, the tobacco sector, led by a few multinational corporations, possesses considerable pricing power and strategically adjusts excise taxes. In imperfectly competitive markets, the allocation of tax burdens differs based on market factors and demand [13]. A prevalent industry strategy is differential tax shifting, in which taxes are selectively transferred across brand categories. Premium brands, whose consumers are less sensitive to price changes, frequently experience over-shifting, where price increases exceed the tax rise to optimize profits. However, cheaper brands encounter under-shifting, as sellers absorb a portion of the tax to keep prices low and maintain sales of those affordable cigarettes [14–18].

Vast empirical evidence from many countries confirms heterogeneous industry pricing strategies and excise tax pass-through across market segments. The literature shows that over-/under-shifting of taxes is often applied differently in high-income countries (HIC) versus low-and middle-income (LMIC) countries. In HIC, the tobacco industry mainly acts to widen the price gap between tobacco market segments by increasing the price of premium products more than is required, while keeping products in economy segments affordable by under-shifting tax increases [19–26]. In LMICs, the tobacco industry usually absorbs a great portion of the tax increase in all market segments to expand their markets, thus losing higher profits in the short term. Moreover, in the case of market disturbances, such as a larger share of illicit tobacco products, research suggests that the pricing strategy is switched from over-to under-shifting to maintain prices on a competitive low level [11,27–30].

Montenegro’s tobacco sector is oligopolistic, dominated by two major international companies – Philip Morris International and Japan Tobacco International – alongside a few importers and wholesalers with no domestic production. Pricing data indicate greater price fluctuations among premium brands than among mid-tier and cheapest brands, suggesting segment-specific pricing tactics. Given that excise taxes represent almost 60% of the weighted average retail price of cigarettes (as of 2023), understanding tax pass-through dynamics is important for an effective tobacco control policy.

This research estimates excise tax pass-through in Montenegro’s cigarette market, analyzing how taxation influences pricing, which is one of the key determinants of cigarette consumption. This analysis is particularly important given the lack of similar studies in Montenegro and the broader Eastern European region, filling a critical gap in the understanding of tobacco industry pricing behavior in these markets. A clearer insight into these dynamics can lead to more effective tax policies aimed at decreasing smoking rates while improving public health, increasing excise tax revenues, and reducing the economic cost of tobacco use [6].

## Materials and methods

### Data

The empirical section of this study is based on three different types of data for the period 2010–2022. The initial dataset includes a micro-database that encompasses monthly data on prices and quantities sold per pack for each brand of cigarettes in the market, obtained from the Montenegrin Ministry of Finance Directorate for Issuing Permits for the Production, Processing, and Trade of Tobacco Products. Given that cigarettes account for the largest share of the tobacco market (95.3%, according to [4]), this study focuses exclusively on this specific tobacco product. Since various brands entered and exited the market in the observed period, this data is used to construct an unbalanced panel covering 269 brands over 156 months. From this database, research will use the retail price as the main dependent variable. The price range of the most-sold brand during the observation period was €0.70 to €2.60 (Table 1).

**Table 1:** Prices of most-sold, premium, and cheapest brands, 2010–2022.

Year	The most-sold brand	Price (€)	Premium brand	Price (€)	Cheapest brand	Price (€)
**2010**	Drina denifine	0.7	Marlboro	1.7	Cuba	0.4
**2011**	Drina denifine	1	Marlboro	2	Monte black	0.5
**2012**	Drina denifine	1.2	Marlboro	2.2	Monte black	0.75
**2013**	Code blue	1.3	Marlboro	2.4	York YLB hard pack	1
**2014**	Ronhill wave black	1.5	Marlboro	2.5	York YLB hard pack	1
**2015**	Ronhill wave black	1.6	Marlboro	2.6	Negro	1
**2016**	L&M loft blue	1.8	Marlboro	2.7	Negro	1
**2017**	L&M loft blue	2	Marlboro	3	Negro	1
**2018**	Winston XStyle long blue	2.3	Marlboro	3.4	Code red	1.6
**2019**	Winston XStyle long blue	2.3	Marlboro	3.4	LD red	1.8
**2020**	Winston XStyle long blue	2.4	Marlboro	3.3	LD Club compact blue	2
**2021**	Winston XStyle long blue	2.5	Marlboro	3.4	Una slims gold	2
**2022**	Winston XStyle long blue	2.6	Marlboro	3.5	Fast revolution 8	2.1

Source: Ministry of Finance.

Note: Prices are given in current values.

The micro-database also contains information on brand varieties, enabling the use of brand characteristics in the analysis. Cigarette brands are categorized by nicotine and tar levels and stick size (e.g., long, short, mild, slim). This classification allows an assessment of differences in excise tax pass-through levels, particularly for milder-tasting cigarettes, such as slim cigarettes, which are a popular choice among young people and women in Montenegro [4]. In addition, the research presents excise tax pass-through effects on various segments of the tobacco market. Premium, mid-price, and economy segments are classified based on price ranges of brands given in the database, industry reports, and brand categories identified by retailers. The same segmentation strategy has been utilized in comparable studies [31].

**Fig 1:**
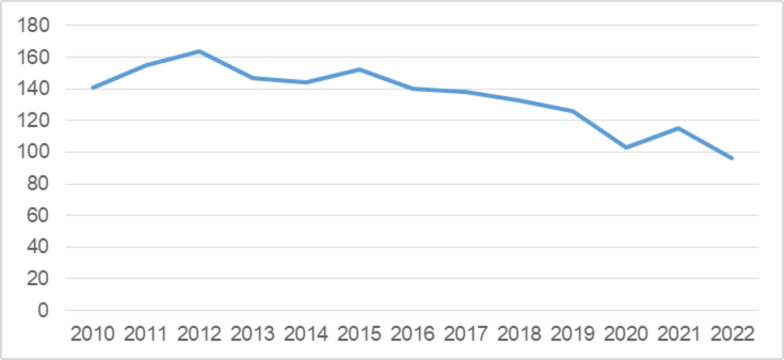
Number of cigarette brands by year 2010–2022. Source: Ministry of Finance, Montenegro.

Overall, the micro-database reports a downward trend in the total number of cigarette brands available in Montenegro, recording a decrease of 47 brands in 2022 compared to 2012 (Fig 1).

The second data source consists of regulatory data extracted from key legislation, including the Law on Excise Duties and the Law on Value-Added Tax (VAT), establishing the rates of specific and ad valorem excise duties and VAT. During the observed period, the specific excise rate increased significantly from €5 per 1,000 sticks of cigarettes in 2010 to €44 per 1,000 sticks in 2022, while the ad valorem tax decreased by nine percentage points (Table 2). This change in excise tax brought Montenegro to the total tax share of 78.02% of weighted average price of cigarette (WAPC) in 2022, surpassing the World Health Organization’s minimum tax burden standard on cigarette retail prices (75 percent). Price changes predominantly occur when tax changes are implemented, with only slight variation in other periods.

**Table 2:** The excise tax calendar 2010–2022.

Period	Specific excise (€ per 1,000 sticks)	Ad valorem excise tax (% of retail price)	VAT (%)
**Jan 2010–Dec 2010**	5	35	17
**Jan 2011–Mar 2012**	10	37	17
**Apr 2012–Jul 2013**	15	36	17
**Aug 2013–Oct 2014**	17.5	35	19
**Nov 2014–Mar 2015**	19	35	19
**Apr 2015–Mar 2016**	20	34	19
**Apr 2016–Mar 2017**	22	32	19
**Apr 2017–Jul 2017**	24	33	19
**Aug 2017–Dec 2017**	30	32	19
**Jan 2018–Aug 2018**	40	32	21
**Sep 2018–Dec 2019**	30	32	21
**Jan 2020–Dec 2020**	33.5	30.5	21
**Jan 2021–Dec 2021**	37	29	21
**Jan 2022–May 2022**	40.5	27.5	21
**Jun 2022–Dec 2022**	44	26	21

Source: Law on Excise Taxes, Law on Value Added Tax, Ministry of Finance.

The third data set contains macroeconomic control variables, including national-level monthly time-series data on average wages and the consumer price index (CPI), obtained from the Statistical Office of Montenegro (Monstat).

The expected retail price, the primary independent variable, was constructed using monthly price data for each brand, changes in excise taxes, and the CPI. This variable assumes that any changes in excise taxes are fully passed on to consumers in each period. The assumption of full pass-through for this variable is used only to construct a counterfactual benchmark*.* Since the Montenegrin market is not perfectly competitive, a difference between the expected price and the actual retail price for some price categories of brands is expected, indicating the extent of tax under- or over-shifting.

The construction of this variable starts with the retail price of each brand for the month it was first entered into the database. This initial price is adjusted in subsequent months to account for inflation and any tax changes. More specifically, the expected price was derived through the following three steps:

1. First, having information on retail price, specific excise (SE), ad valorem excise tax (ADV), and VAT, amount of net of tax (NOT) is estimated for the brand’s entry price using the following Equation (1):

$$\begin{array}{c} {NOT}_{it=\text{1}}=\;\frac{P_{it=\text{1}}}{\left(\text{1}+{VAT\%}_{t=\text{1}}\right)}-{{SE}_{t=\text{1}}-ADV}_{t=\text{1}} \end{array}$$ (1)

where, *P* denotes the retail price per pack, *i* represents a specific brand in the sample, and *t* corresponds to the observed month.

1. In the second step, the value of NOT for the upcoming months (RNOT) is estimated as a product of *NOT*_*t*−1_ and the monthly CPI. Utilizing RNOT and information on taxes for each month *t*, the expected price *PE*_*it*_ is calculated as indicated in Equation (2):

$$\begin{array}{c} {PE}_{it}=({RNOT}_{it}+{SE}_{it}+{ADV}_{it})\times {(\text{1}+VAT\%}_{t}) \end{array}$$ (2)

1. In the third step, the minimum excise tax (ME) requirement was incorporated, as provided for in the Law on Excise Taxes. This regulation stipulates that the ME shall equal 100% of the total excise calculated for the WAPC for the corresponding period. If the sum of specific and ad valorem excise taxes is less than the ME threshold, the ME is applied instead. For brands subject to this adjustment, the expected price is calculated as follows:

$$\begin{array}{c} {PE}_{it}=({RNOT}_{it}+{ME}_{it})\times \left(\text{1}+{VAT\%}_{t}\right) \end{array}$$ (3)

### Empirical approach

Building on existing research on the impact of taxes on tobacco product prices [11,32], this study examines this relationship across various market segments using panel fixed-effects models. To account for unobserved, time-invariant differences in prices across segments, brand fixed effects were used. Monthly time-fixed effects were also included to control for common shocks affecting cigarette prices, such as the enforcement of smoking bans and seasonal or monthly fluctuations. To determine the most appropriate model among OLS, fixed effects, and random effects, a series of diagnostic tests is performed, including the Breusch-Pagan Lagrangian multiplier test, the Sargan-Hansen test, and the Hausman cluster-robust test. The extended baseline specification is given by equation (4a):


$$\begin{array}{c} P_{it}\;=\;\alpha_{i}\;+\;\beta_{\text{1}}\;{PE}_{it}\;+\;\beta_{\text{2}}\;m_{it}\;+\;\beta_{\text{3}}\;({PE}_{it}\;\times \;m_{it})\;+\;\beta_{\text{4}}\;h_{it}\;+\;\beta_{\text{5}}\;({PE}_{it}\;\times \;h_{it})\;+\;\beta_{\text{6}}\;s_{it}\;+\;\beta_{\text{7}}\;X_{t}\;+\;\lambda_{t}+\;u_{it} \end{array}$$ (4a)

equation (4b) specifically assesses the pricing behaviour of slim cigarettes:


$$\begin{array}{c} P_{it}\;=\;\alpha_{i}\;+\;\gamma_{\text{1}}\;{PE}_{it}\;+\;\gamma_{\text{2}}\;s_{it}\;+\;\gamma_{\text{3}}\;({PE}_{it}\;\times \;s_{it})\;+\;\gamma_{\text{4}}\;X_{t}+\;\lambda_{t}\;+\;u_{it} \end{array}$$ (4b)

Alongside these extended specifications, two restricted baseline models are estimated, focusing on the core interaction structure and excluding control variables.

In equations (4a) and (4b) *Pit* donates retail price, *PE*_*it*_ is the expected price constructed under the assumption of the full pass-through, and *m*_*it*_ and *h*_*it*_ are dummy variables representing the mid-price and high-end (premium) cigarette market segment, respectively, with the economy (low-price) segment serving as the reference category. A dummy variable, *s*_*it*_ identifies slim cigarettes. *X*_*t*_ represents macroeconomic variables, such as the national-level average monthly net wage. Additionally, index *i* refers to the cigarette brand (*i* = 1,…269) and *t* indicates monthly observations (*t* = 1,...156).

The coefficient β_1_ in equation (4a), measures tax pass-through in the reference (economy) segment. A value equal to 1 implies full pass-through, values below 1 indicate under-shifting, while values above 1 indicates over-shifting. The interaction terms β_3_ and β_5_ capture differential pass-through in the mid-price and premium segments, so that total pass-through equals β_1_ + β_3_ for the mid-price segment and β_1_ + β_5_ for the premium segment. Similarly, in equation (4b), γ_3_ captures whether slim cigarettes exhibit a different pass-through compared to non-slim products, with total pass-through for slim cigarettes given by γ_1_ + γ_3._

Besides this model, research also applies the quantile regression approach to unbalanced panel data to obtain more accurate and detailed insights into the effect of tax increases on cigarette prices, which represents the advantage of this study. This approach enables a flexible analysis by treating the total price range of all brands as the dependent variable, in contrast to focusing only on the predicted mean value, like in simple regression analysis. It allows estimation of how tax pass-through varies across different price levels, highlighting possible differences in pricing strategies throughout the price distribution. The advantage of this methodology is robustness to outliers and estimation of separate regression lines for each quantile of price distribution. Also, what is important to note is that, as price quantiles are exogenously defined, subjectivity is ruled out, which could be present in any other criteria of price segmentation.

Similar to the existing literature [24,33], the analysis uses 11 quantiles. More precisely, the following points of the price distribution are used: 0.05, 0.15, 0.25, 0.35, 0.45, 0.5, 0.55, 0.65, 0.75, 0.85, and 0.95, representing the 5^th^ percentile (cheapest price brands) to the 95^th^ percentile (the most expensive). Price percentiles are computed using the number of packs sold as weights. The model is estimated as shown in Equation (5):


$$\begin{array}{c} P_{it}=\delta_{\text{0}}+\delta_{\text{1}}{PE}_{it}+\delta_{\text{2}}X_{t}+\varepsilon_{it} \end{array}$$ (5)

In this context, *X*_*t*_ refers to a vector of control variables, including a dummy variable set to 1 if a brand is classified as “slims” and 0 otherwise. National-level average monthly wages are also included as a control variable. The coefficient δ_1_ reflects the extent of the tax pass-through cigarette prices: δ_1_ = 1 indicates full pass-through, δ_1_ < 1 shows tax undershifting (meaning less than full pass-through to prices), and δ₁ > 1 indicates tax overshifting (where prices rise by more than the tax amount). Standard errors are calculated using a clustered bootstrap method to deal with potential challenges like heteroscedasticity and serial autocorrelation.

## Results

### Descriptive statistics

In nominal terms, cigarette prices in Montenegro have shown a clear upward trend overall and across market segments over the observed period. Still, the prices remained among the lowest in the EU – in 2022, the weighted-average prices (WAPC) amounted to €2.73 (S1 Table), which is substantially below the EU average of €6.02 [10]. The WAPC is calculated as a sales-weighted measure that accounts for individual brands’ market shares, as reflected in the number of packs sold.

**Fig 2:**
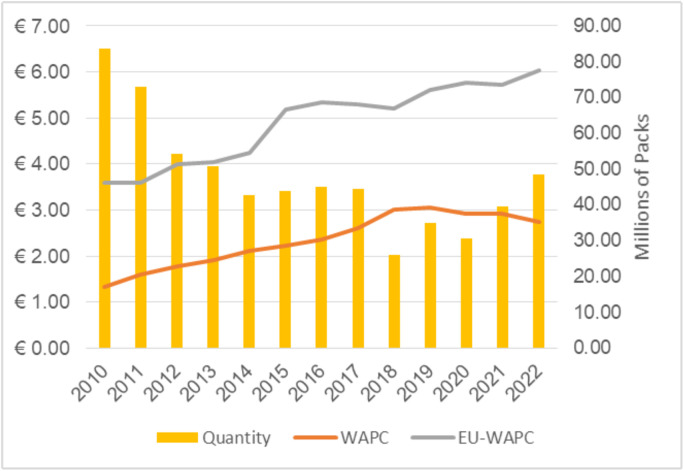
Real WAPC in Montenegro and the EU and the quantity of cigarette packs sold in Montenegro, 2010–2022. Note: WAPC represents weighted-average prices in Montenegro, and EU-WAPC represents weighted-average prices in the EU. Both WAPCs are reported in 2022 constant euros per pack. Quantities are measured in millions of packs. EU-WAPC are sourced from Euromonitor [10] and converted to real euro values using the Eurostat Harmonized Index of Consumer Prices [35] and USD/EUR exchange rate data from the International Monetary Fund [36].

Measured in real terms (constant 2022 euros), the WAPC in Montenegro reached its peak in 2019 and has since shown a slight decline. This indicates that price increases did not keep up with inflation, unlike the weighted-average prices in the EU (EU-WAPC), which followed a more stable upward trend. Throughout the entire period, prices in Montenegro remained significantly lower than the EU average (Fig 2). The volume of cigarettes sold dropped sharply in 2018, coinciding with a notable rise in illicit trade, which likely contributed to the reduction in legal market activity [34].

**Fig 3:**
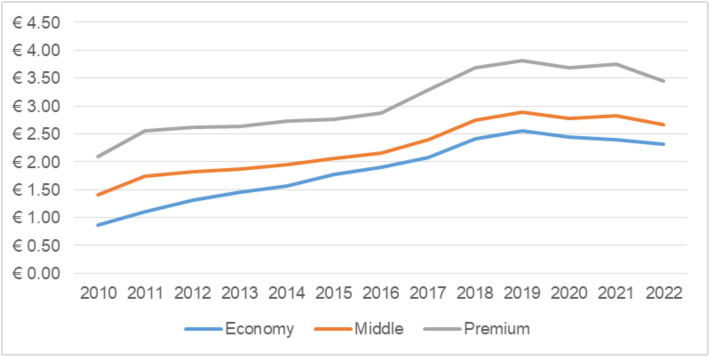
Real WAPC across market segments. Note: WAPC across market segments is reported in 2022 constant euros per pack.

A closer look at the different market segments in Montenegro (Fig 3) confirms the previous pattern, where real WAPC levels across segments exhibited a broadly similar upward trend until 2019, followed by a modest decline thereafter.

**Fig 4:**
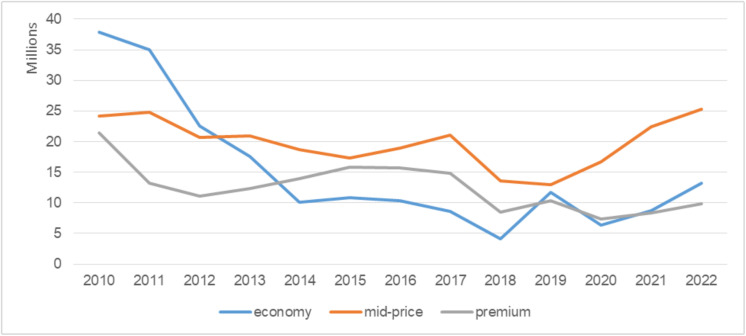
Quantity of cigarettes sold across market segments. Note: Quantity of cigarettes sold across market segments is measured in millions of packs.

Over the observed period, most market activity remains concentrated in the economy and mid-price segments (Fig 4).

This distribution indicates sustained consumer demand for mid-range and economy cigarettes, suggesting price sensitivity among a significant share of consumers (Table 3).

**Table 3:** WAPC and share of cigarette sales by market segment, 2020–2022.

	2020		2021		2022	
	WAPC (€)	Share	WAPC (€)	Share	WAPC (€)	Share
**Economy**	2.11	20.93%	2.12	22.14%	2.32	27.40%
**Mid-price**	2.41	54.76%	2.50	56.63%	2.70	52.34%
**Premium**	3.18	24.31%	3.32	21.23%	3.51	20.26%

Source: Authors’ calculations.

Note: WAPCs are reported by market segment in nominal euros per cigarette pack.

To illustrate the distribution of cigarette prices, Table 4 reports sales-weighted price percentiles for 2020–2022, computed using the number of packs sold as weights. Corresponding results for earlier years are presented in the Supporting information (S1 Table).

**Table 4:** Sales-weighted distribution of cigarette prices, 2020–2022.

Year	WAPC(95% CI)	Min	Q_1_	Median	Q_3_	Max
**2020**	2.53 (2.51–2.55)	1.90	2.30	2.40	2.50	5.00
**2021**	2.59 (2.57–2.61)	1.70	2.30	2.50	2.60	5.00
**2022**	2.73 (2.70–2.76)	2.00	2.40	2.70	2.80	5.00

Source: Authors’ calculations.

Note: WAPC denotes the weighted average price per pack; values in parentheses indicate 95% confidence intervals. Q_1_, Q_2_ and Q_3_ denote the 25^th^, 50^th^ (median), and 75^th^ percentiles of prices. All percentiles are computed using the number of packs sold as weights. Prices are expressed in nominal euros per cigarette pack.

The results indicate a gradual upward shift in the price distribution over time, with the median increasing from €2.40 in 2020 to €2.70 in 2022. At the same time, the interquartile range of just €0.40 in 2022 demonstrates that 50% of the total market volume is concentrated within a very narrow price band. This high density of consumption between €2.40 and €2.80 signals a significant homogenization of consumer demand.

The analysis also examined brand dynamics, including the entry of new brands, the re-entry of previously exited ones, and the exit of existing products during the observed period. Between 2013 and 2019, a clear pattern emerged: more brands exited the market than entered, especially within the economy segment (S2 and S3 Tables). Most of these brands held only marginal market shares. Notably, a small group of brands (31 in total) was found to repeatedly enter and exit the market during this period, maintaining a relatively stable, though limited, presence across all three price segments. Additionally, it was also interesting to assess the impact of brand varieties, specifically slim cigarettes. These products are mainly found in the first two tiers (economy and mid-price), with a noticeable increase in their share in the economy tier, from 32 percent in 2010–54 percent in 2022 (S4 Table). The same conclusion can be drawn when data are divided into quantiles, with the lowest share in the highest quantiles (S5 Table). It is important to notice that slim cigarettes are known as the most attractive to promote among specific groups, such as women and youth, the most common industry strategy.

### Regression results

Both empirical approaches, panel fixed effects and quantile regression, show a pattern that reflects a deliberate same pricing strategy: the industry keeps the cheapest products relatively affordable, likely to retain price-sensitive consumers, while raising prices more significantly on premium brands. Therefore, the estimates reinforce the consistency of tax pass-through behaviours.

To provide more precise and consistent results, the different tobacco industry pricing strategies were estimated using four models (Table 5). Model 1 includes only the expected price and its interactions with price segments as independent variables, providing evidence that under-shifting is present in the economy segment, over-shifting occurs in the premium tier, and in the mid-price segment, there is almost complete tax pass-through. The Model 2 is extended with additional control variables to assess parameter consistency. There is a slightly greater under-shifting in the economy and mid-price tiers, with slim cigarettes being cheaper compared to other types of these products (Model 2). Furthermore, the interaction between slim cigarettes and the expected price (Models 3 and 4) suggests that slim products show a weaker price response to tax changes, implying that their prices increase by less than the corresponding excise tax increase.

**Table 5:** Pass-through of taxes by market tiers.

Variables	Model 1	Model 2	Model 3	Model 4
**Expected price**	0.956***	0.931***	1.069***	1.049***
	(0.004)	(0.004)	(0.002)	(0.003)
**Mid-price**	0.072***	0.082***		
	(0.010)	(0.010)		
**Mid-price X Expected price**	0.034***	0.029***		
	(0.005)	(0.005)		
**Premium**	−0.113***	−0.108***		
	(0.011)	(0.011)		
**Premium X Expected price**	0.184***	0.183***		
	(0.005)	(0.005)		
**Slims X Expected price**			−0.107***	−0.109***
			(0.005)	(0.005)
**Slims**		−0.498***	−0.388***	−0.347***
		(0.111)	(0.125)	(0.124)
**Wage**		0.290***		0.222***
		(0.018)		(0.020)
**Constant**	−0.029*	−1.909***	−0.156***	−1.592***
	(0.015)	(0.120)	(0.016)	(0.133)

Source: Authors’ calculations; ***p < 0.01, **p < 0.05, *p < 0.10.

Panel quantile analysis (Table 6) also considers variations in models: Model 1 (only expected price) and Model 2 (incorporating control variables). The first two quantiles in Model 1 show under-shifting, being slightly under the full tax pass-through: 0.922 (Q_5_) and 0.978 (Q_15_). On the other side, the third quantile is characterized by the full pass-through, which is transitioning to over-shifting on the higher quantile levels. Similar results are found in Model 2, indicating a more pronounced under-shifting in lower quantiles. Going further in the results explanations and analysis, all quantiles above the median demonstrate a clear strategy to increase prices more compared to the increase in tax. Aligning with earlier results, it has been confirmed that slim cigarettes – already priced lower than other types – experience smaller price increases relative to excise tax hikes, reflecting consistent under-shifting by the industry.

**Table 6:** Pass-through of taxes, by quantile regression.

	Model 1		Model 2					
Quantiles	Expected price (€)	St. dev	Expected price (€)	St. dev	Slims	St. dev	Wage	St. dev
**Q**_**5**_	0.922***	(0.021)	0.856***	(0.027)	−0.762**	(0.348)	0.808***	(0.122)
**Q**_**15**_	0.978***	(0.059)	0.929***	(0.021)	−0.710***	(0.236)	0.552***	(0.098)
**Q**_**25**_	0.999***	(0.023)	0.955***	(0.020)	−0.692***	(0.203)	0.458***	(0.096)
**Q**_**35**_	1.015***	(0.022)	0.980***	(0.019)	−0.673***	(0.179)	0.368***	(0.099)
**Q**_**45**_	1.034***	(0.022)	1.009***	(0.019)	−0.653***	(0.165)	0.266**	(0.106)
**Q**_**50**_	1.042***	(0.021)	1.022***	(0.019)	−0.643***	(0.164)	0.221**	(0.107)
**Q**_**55**_	1.051***	(0.021)	1.037***	(0.018)	−0.633***	(0.168)	0.166	(0.111)
**Q**_**65**_	1.069***	(0.021)	1.064***	(0.018)	−0.613***	(0.187)	0.069	(0.121)
**Q**_**75**_	1.090***	(0.021)	1.098***	(0.018)	−0.589***	(0.225)	−0.050	(0.133)
**Q**_**85**_	1.115***	(0.022)	1.141***	(0.018)	−0.558*	(0.287)	−0.203	(0.158)
**Q**_**95**_	1.167***	(0.023)	1.226***	(0.023)	−0.496	(0.431)	−0.506**	(0.199)
**location**	1.045***	(0.004)	1.025***	(0.019)	−0.641***	(0.165)	0.208*	(0.113)
**scale**	0.054***	(0.004)	0.084***	(0.006)	0.061	(0.161)	−0.300***	(0.048)

Source: Authors’ calculations; ***p < 0.01, **p < 0.05, *p < 0.10.

Note: Bootstrapped standard errors given in parentheses (1,000 replications).

To check the robustness of the results, analysis was also conducted separately for the period 2018–2021, characterized by a high share of the illicit market [34], the COVID-19 pandemic, and the introduction of the indoor smoking ban in 2019 [37], and for the period outside this interval (S6 Table). Results show stronger under-shifting during 2018–2021, particularly in the lower price quantiles, compared to other periods.

S1 Graph in the Supporting information shows that the quantile coefficient is outside the OLS confidence interval and that there are significant differences between the quantile and OLS coefficients (when the variable is significant in the scale function (Table 6), its coefficients vary across quantiles). The test of equality of slope estimates across various quantiles is given in S7 Table.

## Discussion and conclusion

This study’s findings provide new evidence that tobacco industry pricing responses to excise tax increases are heterogeneous across product segments, with systematic undershifting observed in cheaper cigarette products and overshifting in premium products. This pattern in Montenegro closely mirrors the segment-specific pass-through documented in high-income countries and extends this literature by demonstrating that similar pricing strategies are also present in a low- and middle-income country context.

In HIC settings, a robust body of research has documented results similar to those observed in the Montenegro study – heterogeneous pass-through across the price distribution. Study in the UK market [24], using panel data quantile regression, finds that increases in specific tobacco taxes were passed on to consumers at rates above the tax increase for mid- and high-priced cigarettes, while cheaper products saw comparatively lower pass-through, suggesting industry strategies to protect low-priced segments. An updated analysis on the same market using small retailer data and the same methodology [38], confirms heterogeneous tax pass-through across the price distribution, with overshifting most pronounced at higher price quantiles and reduced pass-through at the bottom of the distribution.

A number of studies in HIC context have observed comparable outcomes across different market segments, consistent with our study’s findings. In 2010, New Zealand implemented annual tobacco excise tax increases, and a study was conducted to examine whether retailers followed recommended retail prices and if these reflected the full tax rise [21]. Findings show that budget brands experienced smaller price increases than premium and mainstream brands, suggesting undershifting to cheaper products, which may undermine smoking cessation and hinder taxation goals. A recent systematic review synthesizing this evidence concludes that differential pricing and selective tax absorption are common industry responses to excise tax increases [39]. This results in an increasing price gap between premium and budget cigarettes as well as a wider price range available within each price segment [19,40].

The incomplete pass-through pattern is broadly consistent with findings from LMIC contexts: in Colombia, overshifting occurred for budget and premium brands while mid-priced brands were undershifted [25] and in Mexico, overall excise tax increases were moderately overshifted, but ultra-low-priced cigarettes experienced smaller relative price increases [26]. Similarly, studies from Indonesia report incomplete pass-through of tiered excise taxes across multiple product categories, based on fixed and random effects regressions [11,27], highlighting that heterogeneous pass-through across price segments is a consistent feature even across different LMIC markets.

Considering product characteristics and consumer targeting, the industry appears to deliberately keep certain products, such as slim cigarettes preferred by women, relatively affordable, which is consistent with undershifting in these segments [41–42]. Empirical data from Montenegro [4] show that 84.8% of smokers who consume exclusively slim cigarettes are women. That said, it is completely logical for tobacco companies to keep these products very affordable in the Montenegro tobacco market for their focus groups.

This study contributes to tax pass-through prices literature by showing how firm pricing strategies respond to tax increases in a fully import-based, highly concentrated oligopolistic market. Montenegro’s persistent excise tax growth in the context of EU accession enables industry to anticipate fiscal tightening and systematically protect price-sensitive segments, resulting in stable under-shifting of the cheapest brands. The consistently lower pass-through for slim cigarettes further demonstrates how product differentiation is used to manage tax burdens. Moreover, during the period of high illicit trade in 2018–2021, when the illegal market peaked in 2019 and subsequently declined following strengthened enforcement [34], under-shifting in lower price quantiles intensified, revealing how enforcement capacity constrains pricing responses. Together, these findings demonstrate that tax pass-through is shaped by the interaction of tax changes, market structure, product differentiation, and institutional effectiveness.

Although the findings offer important insights into industry pricing strategies, one methodological limitation relates to the construction of the counterfactual expected price. Following established approaches [24,33], expected prices are constructed from observed baseline prices, tax parameters, and inflation as a counterfactual benchmark under full pass-through. Since baseline prices reflect past firm decisions, strategic pricing embedded in the initial price may be carried forward into the benchmark, introducing endogeneity and shifting the reference point used to measure pass-through, potentially affecting the precision of the estimates. In addition, firms may adjust prices in anticipation of tax changes, which could influence the timing of observed pass-through. However, administrative data indicate that most price adjustments occur in months when excise increases take effect, suggesting that anticipatory pricing is limited in this context.

Raising excise taxes remains one of the most impactful strategies for reducing tobacco use, particularly in countries like Montenegro, where cigarette prices are still considerably low. The Government has made success with tax increases on a semi-annual basis since 2022, reaching the EU threshold in January 2025. Achieving the full public health impact of taxation, therefore, requires consistent and sufficiently strong tax increases that are designed and calibrated with consideration of heterogeneous pricing responses, limiting the scope for strategic absorption in lower-priced products. This will support sustained reductions in smoking prevalence and consumption, alongside growth in excise tax revenue.

Moreover, as the industry is using design and promotion to attract customers and make products more appealing, the Government has to consider including amendments in the Law on Limiting use of tobacco, related to the ban of flavoured products such as menthols, restriction of package colours uses, which can create false perceptions of reduced harm [43–44]. Given evidence that pricing responses differ across product types, particularly slim cigarettes, strengthening the regulation of packaging and product characteristics would complement fiscal measures by reducing the scope for differentiated pricing strategies that allow manufacturers to moderate effective tax transmission in targeted product categories. To cope with the problem of the cigarette’s packs design, cigarette packaging should be made as unattractive and uniform as possible, which means transition to adopting plain or standardized packaging. This action would allow Montenegro to meet Article 11 of the WHO Framework Convention on Tobacco Control.

## Supporting information

S1 Table Quantiles of price paid per pack and quantity sold, 2010–2019. Note: WAPC denotes the weighted average price per packs; values in parentheses indicate 95% confidence intervals. Q_1_, Q_2_ and Q_3_ denote the 25^th^, 50^th^ (median), and 75^th^ percentiles of prices. All percentiles are computed using the number of packs sold as weights. Prices are expressed in nominal euros per cigarette pack.
(PDF)

S2 Table Number of new brands entering the market by year, tiers, and brand variants. Source: Authors’ calculations.
(PDF)

S3 Table Number of brands exiting the market by year, tiers, and brand variants. Source: Authors’ calculations.
(PDF)

S4 Table Shares of slim cigarettes sold by segments, 2010–2022. Source: Authors’ calculations. Note: Percentages are given within segments.
(PDF)

S5 Table Shares of slim cigarettes sold by quantiles, 2010–2022. Source: Authors’ calculations. Note: The data are divided by 11 quantiles (12 quantile bands) and given within quantile bands.
(PDF)

S6 Table Pass-through of taxes by quantile regression, encompassing two different time intervals. Source: Authors’ calculations. Note: Bootstrapped standard errors given in parentheses (1,000 replications).
(PDF)

S7 Table Test of equality of slope estimates across various quantiles. Source: Authors’ calculations.
(PDF)

S1 Graph Quantile regression of expected price coefficients. (TIF)
