# Supplementary material for: Pricing strategies of the tobacco companies in response to cigarette excise tax increases in Montenegro
Source: PLoS One. 2026 Jun 2;21(6):e0335670. doi: 10.1371/journal.pone.0335670 (PMC13229352; doi:10.1371/journal.pone.0335670)
Supplement: S1 Table — Note: WAPC denotes the weighted average price per packs; values in parentheses indicate 95% confidence intervals. Q1, Q2 and Q3 denote the 25th, 50th (median), and 75th percentiles of prices. All percentiles are computed using the number of packs sold as weights. Prices are expressed in nominal euros per cigarette pack. (PDF) [file pone.0335670.s001.pdf]

Year	2010	2011
**WAPC (95% CI)**	0.99 (0.97–1.01)	1.22 (1.20–1.24)
**Min**	0.4	0.5
**Q_1**	0.65	0.85
**Q_2 (Median)**	0.85	1.1
**Q_3**	1.3	1.5
**Max**	4.5	4.9
**Year**	**2012**	**2013**
**WAPC (95% CI)**	1.42 (1.40–1.44)	1.56 (1.54–1.57)
**Min**	0.5	0.75
**Q_1**	1.1	1.2
**Q_2 (Median)**	1.3	1.5
**Q_3**	1.7	1.7
**Max**	4.9	4.7
**Year**	**2014**	**2015**
**WAPC (95% CI)**	1.72 (1.70–1.74)	1.84 (1.82–1.86)
**Min**	0.8	1
**Q_1**	1.4	1.5
**Q_2 (Median)**	1.7	1.7
**Q_3**	1.9	2
**Max**	4.9	4.9
**Year**	**2016**	**2017**
**WAPC (95% CI)**	1.93 (1.92–1.95)	1.93 (1.92–1.95)
**Min**	1	1
**Q_1**	1.7	1.7
**Q_2 (Median)**	1.8	1.8
**Q_3**	2	2
**Max**	3	3
**Year**	**2018**	**2019**
**WAPC (95% CI)**	2.59 (2.56–2.61)	2.64 (2.62–2.67)
**Min**	1.6	1.6
**Q_1**	2.3	2.3
**Q_2 (Median)**	2.4	2.5
**Q_3**	3	2.8
**Max**	4	4
