# Supplementary material for: Pricing strategies of the tobacco companies in response to cigarette excise tax increases in Montenegro
Source: PLoS One. 2026 Jun 2;21(6):e0335670. doi: 10.1371/journal.pone.0335670 (PMC13229352; doi:10.1371/journal.pone.0335670)
Supplement: S2 Table — Source: Authors’ calculations. (PDF) [file pone.0335670.s002.pdf]

	**Economy**	**Middle**	**Premium**	**Slims**
**2011**	10	0	7	0
**2012**	6	5	8	5
**2013**	2	12	4	4
**2014**	2	12	4	4
**2015**	14	5	2	4
**2016**	3	3	3	1
**2017**	5	6	4	7
**2018**	5	5	2	6
**2019**	8	2	0	2
**2020**	2	0	0	2
**2021**	9	13	1	7
**2022**	0	2	3	0
**Total**	**66**	**65**	**38**	**42**
