# Supplementary material for: Pricing strategies of the tobacco companies in response to cigarette excise tax increases in Montenegro
Source: PLoS One. 2026 Jun 2;21(6):e0335670. doi: 10.1371/journal.pone.0335670 (PMC13229352; doi:10.1371/journal.pone.0335670)
Supplement: S3 Table — Source: Authors’ calculations. (PDF) [file pone.0335670.s003.pdf]

	Economy	Middle	Premium	Slims
**2010**	0	4	1	2
**2011**	4	4	2	1
**2012**	11	3	3	1
**2013**	7	14	9	4
**2014**	8	4	1	7
**2015**	16	3	2	4
**2016**	10	4	3	3
**2017**	9	5	3	1
**2018**	5	8	4	5
**2019**	10	17	3	9
**2020**	2	4	3	2
**2021**	16	4	3	10
**Total**	**98**	**74**	**37**	**49**
