# Supplementary material for: Pricing strategies of the tobacco companies in response to cigarette excise tax increases in Montenegro
Source: PLoS One. 2026 Jun 2;21(6):e0335670. doi: 10.1371/journal.pone.0335670 (PMC13229352; doi:10.1371/journal.pone.0335670)
Supplement: S4 Table — Source: Authors’ calculations. Note: Percentages are given within segments. (PDF) [file pone.0335670.s004.pdf]

		Slim cigarettes		
		**Economy**	**Mid-price**	**Premium**
**2010**	%	31.85	21.19	18.69
	CI	(24.23 - 39.47)	(16.61 - 25.77)	(14.12 - 23.25)
**2011**	%	34.91	30.56	4.73
	CI	(27.61 - 42.21)	(25.83 - 35.29)	(2.78 - 6.68)
**2012**	%	21.21	28.47	4.67
	CI	(15.95 - 26.48)	(23.94 - 33.00)	(2.82 - 6.53)
**2013**	%	18.17	29.86	4.74
	CI	(13.38 - 22.97)	(25.07 - 34.66)	(2.93 - 6.55)
**2014**	%	23.85	30.03	4.75
	CI	(18.09 - 29.62)	(25.09 - 34.97)	(3.12 - 6.38)
**2015**	%	18.06	33.63	6.34
	CI	(13.13 - 22.99)	(27.06 - 40.19)	(4.40 - 8.27)
**2016**	%	16.94	34.12	3.35
	CI	(11.66 - 22.21)	(28.20 - 40.05)	(2.19 - 4.50)
**2017**	%	23.52	34.10	4.78
	CI	(16.58 - 30.46)	(28.91 - 39.29)	(3.09 - 6.47)
**2018**	%	38.63	37.88	6.05
	CI	(27.41 - 49.85)	(31.27 - 44.48)	(3.54 - 8.56)
**2019**	%	37.06	32.08	16.66
	CI	(25.99 - 48.13)	(24.87 - 39.28)	(12.15 - 21.18)
**2020**	%	46.40	32.38	5.16
	CI	(36.03 - 56.77)	(23.87 - 40.88)	(3.45 - 6.87)
**2021**	%	53.55	30.67	5.38
	CI	(42.10 - 65.00)	(22.38 - 38.97)	(3.48 - 7.28)
**2022**	%	54.08	29.08	2.28
	CI	(42.01 - 66.16)	(20.57 - 37.58)	(1.32 - 3.24)
