# Supplementary material for: Pricing strategies of the tobacco companies in response to cigarette excise tax increases in Montenegro
Source: PLoS One. 2026 Jun 2;21(6):e0335670. doi: 10.1371/journal.pone.0335670 (PMC13229352; doi:10.1371/journal.pone.0335670)
Supplement: S5 Table — Source: Authors’ calculations. Note: The data are divided by 11 quantiles (12 quantile bands) and given within quantile bands. (PDF) [file pone.0335670.s005.pdf]

	**Slim cigarettes**	
**Quantiles**	**Percent**	**CI**
**≤ Q_5**	22.72	(18.44-27.00)
**Q_5 - Q_{15}**	25.98	(23.61-28.34)
**Q_{15} - Q_{25}**	19.57	(17.61-21.54)
**Q_{25} - Q_{35}**	25.59	(24.18-27.00)
**Q_{35} - Q_{45}**	24.67	(22.88-26.46)
**Q_{45} - Q_{50}**	15.13	(13.98-17.25)
**Q_{50} - Q_{55}**	20.25	(16.15-20.35)
**Q_{55} - Q_{65}**	21.69	(20.16-23.22)
**Q_{65} - Q_{75}**	32.03	(29.93-34.14)
**Q_{75} - Q_{85}**	26.95	(24.56-30.34)
**Q_{85} - Q_{95}**	15.61	(14.08-17.14)
**≥ Q_{95}**	5.11	(3.96-6.26)
