# Supplementary material for: Pricing strategies of the tobacco companies in response to cigarette excise tax increases in Montenegro
Source: PLoS One. 2026 Jun 2;21(6):e0335670. doi: 10.1371/journal.pone.0335670 (PMC13229352; doi:10.1371/journal.pone.0335670)
Supplement: S6 Table — Source: Authors’ calculations. Note: Bootstrapped standard errors given in parentheses (1,000 replications). (PDF) [file pone.0335670.s006.pdf]

	Year (2018–2021)		Year (2010–2017, 2022)	
**Quantiles**	**Expected price (euro)**	**St. dev**	**Expected price (euro)**	**St. dev**
Q_5	0.890***	(0.017)	0.953***	(0.006)
Q_{15}	0.916***	(0.014)	0.973***	(0.004)
Q_{25}	0.946***	(0.011)	0.982***	(0.003)
Q_{35}	0.955***	(0.010)	0.990***	(0.003)
Q_{45}	0.971***	(0.009)	0.999***	(0.004)
Q_{50}	0.977***	(0.009)	1.004***	(0.004)
Q_{55}	0.992***	(0.008)	1.010***	(0.005)
Q_{65}	1.006***	(0.008)	1.022***	(0.006)
Q_{75}	1.011***	(0.008)	1.032***	(0.008)
Q_{85}	1.018***	(0.008)	1.042***	(0.009)
Q_{95}	1.040***	(0.010)	1.064***	(0.012)
**location**	0.973***	(0.009)	1.007***	(0.005)
**scale**	0.041***	(0.006)	0.028***	(0.004)
