# Supplementary material for: Pricing strategies of the tobacco companies in response to cigarette excise tax increases in Montenegro
Source: PLoS One. 2026 Jun 2;21(6):e0335670. doi: 10.1371/journal.pone.0335670 (PMC13229352; doi:10.1371/journal.pone.0335670)
Supplement: S7 Table — Source: Authors’ calculations. (PDF) [file pone.0335670.s007.pdf]

	Model 1		Model 2	
**Quantile**	**chi2(1)**	**p-value**	**chi2(1)**	**p-value**
**5 versus 15**	177.82	0.00	47.23	0.00
**15 versus 25**	203.3	0.00	47.17	0.00
**25 versus 35**	212.33	0.00	47.35	0.00
**35 versus 45**	210.25	0.00	47.45	0.00
**45 versus 50**	172.19	0.00	45.26	0.00
**50 versus 55**	185.42	0.00	46.24	0.00
**55 versus 65**	199.98	0.00	47.16	0.00
**65 versus 75**	209.68	0.00	47.68	0.00
**75 versus 85**	195	0.00	47.35	0.00
**85 versus 95**	201.09	0.00	47.93	0.00
